# Toxicological evaluation of metal oxide nanoparticles and mixed exposures at low doses using zebra fish and THP1 cell line

**DOI:** 10.1002/tox.22692

**Published:** 2018-12-12

**Authors:** Jasreen Kaur, Madhu Khatri, Sanjeev Puri

**Affiliations:** ^1^ Department of Biotechnology, University Institute of Engineering and Technology (UIET) Panjab University Chandigarh India; ^2^ Centre for Nanoscience and Nanotechnology Panjab University Chandigarh India; ^3^ Wellcome trust/DBT IA Early Career Fellow, Panjab University Chandigarh 160014 India

**Keywords:** apoptosis, genotoxicity, nanotoxicology

## Abstract

Metal and metal oxide nanoparticles are being used in different industries now‐a‐days leading to their unavoidable exposure to humans and animals. In the present study, toxicological testing was done using nanoparticles of copper oxide, cerium oxide and their mixture (1:1 ratio) on zebra fish embryos and THP‐1 cell line. Zebrafish embryos were exposed to 0.01 μg/ml to 50 μg/ml concentrations of dispersed nanoparticles using a 96 well plate and their effects were studied at different hours post fertilization (hpf) i.e. 0 hpf, 24 hpf, 48 hpf, 72 hpf and 96 hpf respectively. Results showed that copper oxide nanoparticles has drastic effects on the morphology and physiology of zebra fish whereas cerium oxide nanoparticles and mixture of these nanoparticles did not show much of the effects. Comparable results were obtained from in vitro study using human monocyte cell line (THP‐1). It is concluded that these nanoparticles may cause toxicological effects to humans and environment.

**Abbreviations**
CuO: copper oxide
DLS: dynamic light scattering
GPX: glutathione peroxidase
HMOX: haem oxigenase
NPs: nanoparticles
ROS: reactive oxygen species
SEM: scanning electron microscopy
SOD: superoxide dismutase

## 1 INTRODUCTION

Nanoparticles (NP), with size ranging from 1 to 100 nm, are being manufactured and used in a wide range of applications like cosmetics, biomedicines, electronics, and environment remediation. This is because of their unique physicochemical properties such as quantum effects, surface area as well as unique electrical, mechanical, and imaging properties.^1^, ^2^ Among these NP, metal oxide NP have a broad range of applications, for example, copper oxide (CuO) NP are used in gas sensors, batteries, catalytic organic transformations, electrocatalysis, photocatalysis, solar cells, fuels, and in wound dressings. Cerium oxide NP are utilized in applications like glass polishing and chemical mechanical polishing, nanomedicine, and fuel additives to reduce soot and to increase efficiency of diesel engines.^3^, ^4^ Although decreasing the size of nanomaterial imparts useful traits, however, they also exhibit a size range which is prone to interact with biomolecules, such as proteins and nucleic acids or organelles and therefore interfere with the biological functions leading to the cell damage.^5^, ^6^ Release of large number of these manufactured NP into the environment is raising concerns regarding their potential toxic effects.^7^, ^8^ NP, when released into the environment, are likely to be exposed to organisms in different ways. As far as the human are concerned, the exposure may occur through inhalation, ingestion, or through dermal exposure. NP released in the atmosphere end up in water bodies and water may serve as a transport medium and a temporary reservoir for NP exposing aquatic organisms at large. Therefore, we intend to assess both the ecotoxicological effects and human toxicity of these nanomaterials.^9^

Previously, in vitro studies have indicated that CuO NP cause genotoxicity and cytotoxicity in peripheral blood and cancer cell lines.^7^, ^10^ Several in vivo studies have also been done using *Drosophila melanogaster* as the model organism, which reported the potential genotoxic risk of CuO NP, such as increase in point mutations, alterations in DNA, and DNA strand breaks.^11^ Similarly, nano‐sized cerium oxide NP were found to cause growth inhibition in *Caenorhabditis elegans*.^12^ Thus, clear guidelines need to be set to ensure safe manufacturing procedures and use of these NP. In various processes that are used for the manufacturing of NP, the NP are released in the environment leading to the mixed exposure of these NP to the workers of that particular industry. Particularly, CuO and cerium oxide NP are released into water bodies through common sources such as wastewater treatment plants and from catalyst industries using CuO‐CeO catalysts.^13^, ^14^ In addition, these NP can get mixed in the aquatic system and might be harmful for aquatic as well as terrestrial life.^15^ Therefore, there is also a need to ensure the safety of these mixed NP (1:1 ratio) in both in vitro and in vivo studies as they might exhibit synergistic effect too. Studies concerning the toxicological effects of mixture of NP are not available till date. Thus, the present study is an attempt to explore the toxic effects of mixed exposure NP.

In vitro models cannot be used ideally for assessing the toxicological effects of NP, because although cellular systems are too simplified to predict the organism level interactions, rodents tend to be expensive, complex, and have many ethical issues. Thereby, the in vivo model used for this study was zebra fish due to various reasons like high fecundity, due to which large number of NP can be tested in a less amount of time. Zebra fish embryos also have transparent body which makes development of body organs easy to monitor. Other features such as low cost, easiness to maintain and breed, well‐characterized developmental stages, and availability of different transgenic lines also increases its ability to access the toxicological effects of NP. Several studies have been conducted to access the toxicity of various NP such as iron oxide NP,^16^ silica,^17^ and many more using zebra fish as the model organism. In previous studies, short‐term exposure of zebra fish embryos to CuO NP at high doses had shown hepatotoxicity and neurotoxicity.^18^ In another study, cerium oxide NP were found to affect the GIT of zebra fishes, therefore proving the toxicity of these NP.^19^

To get the clear view of the toxicological effects of these NP, comparative evaluation needs to be done which emphasizes the effect of these NP on both in vivo and in vitro models. For in vitro studies, THP 1 cell line was used. It is a monocytic cell line which can be differentiated into macrophages and therefore can be used to analyze the phagocytic effects of these NP. In this study, doses ranging from very low to high, that is, 0.01, 0.1, 1, 10, and 50 μg/mL, were used as compared to high doses which are usually used by researchers during toxicological studies of NP.^20^ Higher doses lead to large number of toxicological effects, but it is rare that humans are exposed to such higher concentrations of NP. Therefore, it is mandatory to check the toxic effects of NP at lowest concentrations.

In this study, zebra fish embryos and THP 1 cell line were used to study the toxic effects of CuO NP, cerium oxide NP, and their mixture using 96‐well plates, and then, various toxicological end points were considered. The end points that were taken into account were the morphological and physiological changes in zebra fish embryos, comet assay in both cell lines and single‐cell suspension prepared from embryos, cell viability assay in cell lines, checking the hatching rate of zebra fish embryos, and so forth. The results thus obtained from both in vitro and in vivo studies were compared, and their toxicity was evaluated accordingly.

## 2 MATERIALS AND METHODS

### 2.1 Physicochemical characterization of NP in dry state

The physiological characterization of CuO NP (Sigma (St. Louis, Missouri, USA), 50 nm in size), cerium oxide NP (gifted by Dr Dhimiter Bello, UMass, Lowell), and the mixture of these two NP was done by using scanning electron microscopy (SEM). The surface area of these NP was measured by Brunauer‐Emmett‐Teller (BET) analysis, and crystallinity was checked by using X‐ray diffraction (XRD) method.

### 2.2 Preparation of nanoparticle dispersions

Required amount of the given NP was dissolved in Milli‐Q water to make a stock solution of 1 mg/mL concentration. The solution was sonicated for 20 minutes (50 kJ) in a sonicator (Vibra cell, Newtown, CT). The dilutions of this solution were prepared in Holtfreter's medium (medium used to culture zebra fish embryos) to make final concentrations of 0.01, 0.1, 1, 10, and 50 μg/mL. The suspensions were sonicated for 2 minutes each. These dispersions were finally used for dosing the zebra fish embryos.

In case of in vitro studies, NP dispersions were prepared by stirring the given NP in Milli‐Q water and sonicating it for 2 minutes (50 kJ), and then, final solution of 1 mg/mL was prepared by adding it to RPMI 1640 media supplemented with 10% fetal bovine serum (FBS). Different concentrations of these NP were added to the fresh media to make the final concentrations of 0.01, 0.1, 1, 10, and 50 μg/mL, which were further used for dosing. The dosimetry of cerium oxide and CuO of similar size and surface area using RPMI medium has already been reported.^21^, ^22^

### 2.3 Physicochemical characterization of NP dispersions

The given NP were characterized in liquid form in Holtfreter's medium and RPMI 1640 medium by performing dynamic light scattering (DLS) and zeta potential analysis (by using Zetasizer by Malvern) and also by measuring its pH and conductivity. To confirm its size and shape, SEM analysis was done after drying the dispersions. The concentration of ions, that is, copper and cerium ions, which are being formed in the Holtfreter's medium, was analyzed at a concentration of 50 μg/mL, using inductively coupled plasma mass spectrometry (ICP MS). This procedure generally combines a high‐temperature ICP MS. The ICP source converts the atoms of the elements in the sample to ions. These ions are then separated and detected by the mass spectrometer. Therefore, the dispersions of NP were introduced into the ICP as an aerosol by aspirating it into a nebulizer. The ions from the ICP source were then focused by the electrostatic lenses to the mass spectrometer and then detected by a detector.

### 2.4 In vivo and in vitro experiments

#### 2.4.1 Fish husbandry, embryo collection

Wild‐type zebra fish (AB strain) was raised according to standard breeding protocols (28 ± 0.5°C with 14:10 day/night photoperiod) in a recirculation system. Reverse osmosis (pH 6.5‐7.5) filtered water was supplied to the recirculation system with conductivity of 450‐1000 s/cm. Zebra fish were fed twice daily with live Artemia and a dry flake diet. The development status of zebra fish embryos and larvae were observed with an Inverted Microscope (Nikon, Japan). Zebra fish embryos were obtained from spawning adults in tanks overnight with the sex ratio of 1:1. Embryos were collected within 1 hour after the light was switched on and rinsed in Holtfreter's medium before use.

#### 2.4.2 Cell culture

All the cell culture experiments were performed using human monocytic immortalized cells THP‐1 which were cultured in RPMI 1640 supplemented with 10% FBS. All the media was supplemented with 1% penicillin‐streptomycin. The cells were grown in an incubator at 37°C/5% CO^2^ in 75 cm^2^ flasks, and the media was replaced with fresh media after every 2‐3 days.

#### 2.4.3 Exposure

In in vivo studies, embryos were collected and placed in a Petri dish. They were washed with Holtfreter's medium thrice. Embryos were identified at same development stage (4 hours postfertilization [hpf]) and placed in a 96‐well flat bottom plates using plastic pipette with one embryo in each well. The dead embryos were discarded. Then, 100 μL of Holtfreter's medium containing required concentration of given NP was added to each well. These 96‐well plates were kept in an incubator set at 28°C. The zebra fish embryos were examined every 24 hours till 120 hpf. As a control, the zebra fish embryos were also exposed to copper sulfate and cerium oxide salt at similar concentrations as were used in case of nanomaterials. This was to ensure that the mortality is being caused by CuO or cerium oxide NP and not by copper ions or cerium ions.

In in vitro experiments, 5 × 10^5^ cells/mL was used for dosing. These cells were seeded into 96‐well plates and were differentiated into macrophages by culturing in the medium supplemented with 200 nM PMA (Phorbol 12‐myristate 13‐acetate) for 24 hours. The media was replaced with fresh media without PMA. The cell culture media was replaced with 200 μL of fresh media containing different concentrations of NP, that is, 0.01, 0.1, 1, 10, and 50 μg/mL. After 24 hours, the supernatant was thrown and the cells were washed with serum free RPMI 1640 and it was trypsinized for 5 minutes to detach the cells from the 96‐wells plates. The cell suspension was centrifuged and washed with phosphate‐buffered saline (PBS). The cells were used to access cell viability, DNA damage, apoptosis, and cell proliferation. All experiments were performed in triplicates. Statistical analysis between groups was performed using analysis of variance (anova). Results were considered statistically significant at *P* < 0.05 (notation: *P* < 0.001, ***; *P* < 0.01, **; and *P* < 0.05, *). Results were expressed as mean ± SE.

#### 2.4.4 Hatching, malformation and mortality

Mortality and malformations as toxicity endpoints were studied in embryos daily till 120 hpf. The mortality of embryos was confirmed by examining their movement, heartbeat, and blood circulation via inverted microscope (Nikon, Japan), and the results were recorded every 24 hours till 120 hpf. At 72 hpf, hatching was recorded. The presence of physical abnormalities such as head, yolk sac, tail, and pericardia were recorded at 96 hpf, respectively. Each experiment was performed independently in triplicate.

#### 2.4.5 Cell viability

In case of in vitro studies, the cell viability was evaluated after 24 hours of exposure by trypan blue staining. The staining was done by mixing one part of 0.4% trypan blue with one part cell suspension. The mixture was allowed to incubate for 3 minutes at room temperature, and the unstained (viable) and stained (nonviable) cells were counted after loading the mixture on a hemocytometer. The procedure was performed three times, and a total of 900 cells/experiment were counted. Percent viable cells were calculated as: (%) viable cells = (total number of viable cells per mL of aliquot/total number of cells per mL of aliquot) × 100. All the experiments were performed in triplicates.

#### 2.4.6 Catalase assay

The catalase assay was performed by using catalase assay kit (Cayman, Michigan, USA). The kit works on the principle of assessing the catalase enzyme activity by utilizing the peroxidatic function of catalase. It is based on the reaction of enzyme with methanol in the presence of H_2_O_2_ in optimal concentrations. Ten embryos were taken from the groups treated with different concentrations of the given NP and were homogenized in cold buffer (50 mM potassium phosphate, pH 7 containing 1 mM ethylenediaminetetraacetic acid [EDTA]), and then, the supernatant was collected and used for further experiments. All the experiments were performed according to the protocol given along with kit. The reaction was stopped by adding catalase potassium periodate, and the absorbance was read at 540 nm. The absorbance of standard as a function of final formaldehyde concentration was plotted, and the formaldehyde concentration of the samples was calculated. Then, the catalase activity of the samples was calculated where one unit is defined as the amount of enzyme that will cause the formation of 1.00 nmole of formaldehyde per minute at 25°C. The experiment was performed in duplicates.

#### 2.4.7 DNA damage using comet assay

Embryos were taken from each concentration of the given three NP, and single‐cell suspensions of these embryos were prepared in L‐15 medium (Sigma, St. Louis, Missouri, USA) after treatment of embryos with trypsin containing 1 mM EDTA and mixture of enzymes (collagenase, proteinase K, and hyaluronidase). In case of in vitro studies, cells were taken directly from wells. The comet assay was performed for DNA damage using a commercially available kit (Cell Biolabs, San Diego, CA, USA). The steps were performed according to the protocol described in the kit with one modification, that is, regular microscope slides coated with 1% agarose and dried overnight at room temperature were used instead of slides provided with the kit. The slides were stained with Vista green and viewed using epifluorescence microscopy with a fluorescein isothiocyanate filter. The analysis of cell images was done using CASP software. The percent of DNA in the tail (tail DNA %) was reported as a marker of DNA damage. For each concentration and time point, 100 comets were analyzed and scored. All experiments were performed in triplicate.

#### 2.4.8 Quantitative polymerase chain reaction

Total RNA was isolated from 20 embryos from the groups of zebra fish embryos treated with each concentration at 72 hpf by Trizol method, and cDNA was made by using Verso cDNA synthesis kit (Thermo Scientific). Quantitative polymerase chain reaction (PCR) was performed using SYBR green mastermix for three oxidative stress related genes, that is, superoxide dismutase 1 (SOD1), glutathione peroxidase 1 (GPX1), and haem oxigenase 1 (HMOX1). The housekeeping genes used as internal control were 18s rRNA and glyceraldehyde 3‐phosphate dehydrogenase (Table 1). The primers used for performing Q PCR are given in Supporting Information Table S1. Fold change in gene in treated group compared to the untreated group was calculated using 2^−ΔΔCT^ method.

**Table 1:** Primers used for carrying out qPCR reactions

Gene	Sequence	Product size (bp)	*T*_m_ (°C)
18s rRNA	F‐5′‐TCGCTAGTTGGCATCGTTTATG‐3′ R‐5′‐CGGAGGTTCGAAGACGATCA‐3′	123	53
GAPDH	F‐5′‐GTGGAGTCTACTGGTGTCTTC‐3′ R‐5′‐GTGCAGGAGGCATTGCTTACA‐3′	1331	54
SOD 1	F‐5′‐AGACCTGGGTAATGTGACCG‐3′ R‐5′‐CGGGCTAAGTGCTTTCAGAG‐3′	773	59.4
HMOX 1	F‐5′‐GGAAGAGCTGGACAGAAACG‐3′ R‐5′‐GACAGATCTCCGAGGTAGCG‐3′	1413	60
GPX 1	F‐5′‐GAGGCACAACAGTCAGGGAT‐3′ R‐5′‐TCTCCCATAAGGGACACAGG‐3′	994	59.4

Abbreviations: GAPDH, glyceraldehyde 3‐phosphate dehydrogenase; GPX, glutathione peroxidase; HMOX, haem oxigenase; qPCR, quantitative polymerase chain reaction; SOD, superoxide dismutase.

#### 2.4.9 Apoptosis assay

Single‐cell suspensions were prepared from embryos taken from the ones treated at each concentration at 96 hpf, whereas treated cells were taken directly from in vitro experiments. The cells were washed thrice with PBS and stained with Annexin V using APC Annexin V (BD Pharmingen, India), and the cells were also co‐stained with propidium iodide (Sigma, St. Louis, Missouri, USA). Stained cells were analyzed immediately by flow cytometer (using BD Accuri). All the experiments were performed in triplicates.

#### 2.4.10 MTT assay

The MTT (3‐[4,5‐dimethylthiazol‐2‐yl]‐2,5 diphenyl tetrazolium bromide) assay was performed on the treated cells in in vitro studies, although they were in the medium itself. It was performed using MTT assay kit. The MTT reagent was added to these cells, and the plate was covered and incubated at 37°C/5% CO_2_ for 2 hours. After the incubation, the solubilizaton buffer was added to each well and pipetted up and down to dissolve crystals. The plate was then read at 570 nm. The percent survival of these cells was calculated by the formula: (absorbance of sample/absorbance of control) × 100. All the experiments were performed in triplicates.

#### 2.4.11 Cytokine analysis

In in vitro studies, samples were assayed using an ELISA kit (Thermofisher Scientific, India) according to the manufacturer's instructions. The results were analyzed using EON Biotek illuminator. Cytokine concentrations were calculated using standard curve. The three cytokines included were IL 6, IL 8, and TNF α.

## 3 RESULTS

### 3.1 Characterization of NP

The primary particle sizes of CuO and cerium oxide NP were around 50 nm and 10 nm, respectively, and the surface area through BET analysis is shown in Table 2. The crystallinity of these NP was also checked using XRD analysis (Supporting Information Figure S6). The CuO and cerium oxide NP were found to be crystalline in nature. The peaks were found to be similar to the ones obtained in previous studies. The hydrodynamic sizes in Holtfreter's medium of CuO NP, cerium oxide NP, and the mixture of these NP were found to be increased to 377 ± 23 nm, 336 ± 58 nm, and 493 ± 11 nm, respectively. The size change was further confirmed by SEM images (Supporting Information Figure S1). The zeta potential values for these NP were also in range of −15 to −22 μE. The ICP MS data showed that when CuO NP were mixed in Holtfreter's medium at a concentration of 50 μg/mL, the concentration of copper ions was found to be 1.25 μg/mL. Similarly, when cerium oxide NP were mixed with Holtfreter's medium at same concentration, the concentration of cerium ions were found to be around 2.23 μg/mL. When the mixture of NP was mixed with Holtfreter's medium, the concentration of copper ions and cerium ions was found to be 0.65 μg/mL and 0.12 μg/mL, respectively. The ICP MS data also showed that when salts of copper and cerium were mixed in Holtfreter's medium at a concentration of 50 μg/mL, the concentration of ions was found to be 15.62 μg/mL and around 6.34 μg/mL, respectively. The pH and conductivity of these dispersions were also measured and are shown in Table 2.

**Table 2:** Physical characteristics of dry nanoparticles and dispersions of various nanoparticles

		Dry form							
		BET analysis			Dispersion form				
S. no.	Name of the nanoparticle	Surface area (m^2^/g)	Pore volume (cm^3^/g)	Pore radius (Å)	Size (nm)	PDI	Zeta potential (μE)	pH	Conductivity (μS)
1	Copper oxide nanoparticles	112.12	0.520	86.88	377 ± 23	0.2	−21.1 ± 1.4	8.310	1708
2	Cerium oxide nanoparticles	11.892	0.078	19.105	336 ± 58	0.3	−17.2 ± 0.3	8.360	1316
3	Mixture of copper oxide and cerium oxide nanoparticles	…	…	…	493 ± 11	0.2	−18.2 ± 1.3	8.427	1265

Abbreviations: BET, Brunauer‐Emmett‐Teller; PDI, polydispersity index.

### 3.2 Results of in vivo and in vitro experiments

#### 3.2.1 Mortality rate, hatching rate, and malformations

**Figure 1:**
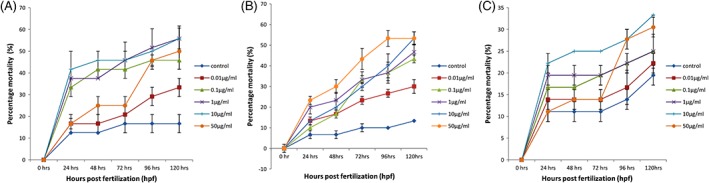
Mortality rate of zebra fish embryos treated with (A) copper oxide nanoparticles, (B) cerium oxide nanoparticles, and (C) mixture of copper oxide and cerium oxide nanoparticles. Data are expressed as means ± SE from three independent experiments. Analysis of variance (α < 0.05) [Color figure can be viewed at wileyonlinelibrary.com]

The mortality rate of zebra fish embryos was observed at different time intervals, and it was found that in case of embryos treated with CuO NP, the mortality rate increased in a dose‐dependent and time‐dependent manner up to 10 μg/mL to 55.83%, but then, it decreased to 50% at the highest concentration, that is, 50 μg/mL. Whereas in case of embryos treated with cerium oxide NP dose‐dependent and time‐dependent increase in mortality rate was observed with the highest mortality of about 53% at the concentration of 50 μg/mL. In the embryos treated with the mixture of these NP, the mortality rate increased up to 10 μg/mL concentrations, that is, 33.3%, but again a decrease was observed in 50 μg/mL concentration to 30% (Figure 1). When these zebra fish embryos were treated with salt‐containing copper ions, it was found that no effect was found on mortality rate of fishes with increasing dose and time of exposure of these NP. The maximum mortality rate was around 10% in comparison to control (no salt), and no malformations were observed. Similarly, when the zebra fish embryos were treated with cerium oxide salt, no significant effect was found on mortality rate of fishes with increasing dose and time of exposure of NP. The maximum mortality rate was only around 5% as compared to control without the appearance of any malformations.

The hatching rate observed 72 hpf in case of zebra fish embryos treated with CuO NP, cerium oxide NP, and the mixture of these NP at highest concentration of 50 μg/mL were found to be 0%, 25%, and 20%, respectively (Supporting Information Figure S2).

**Figure 2:**
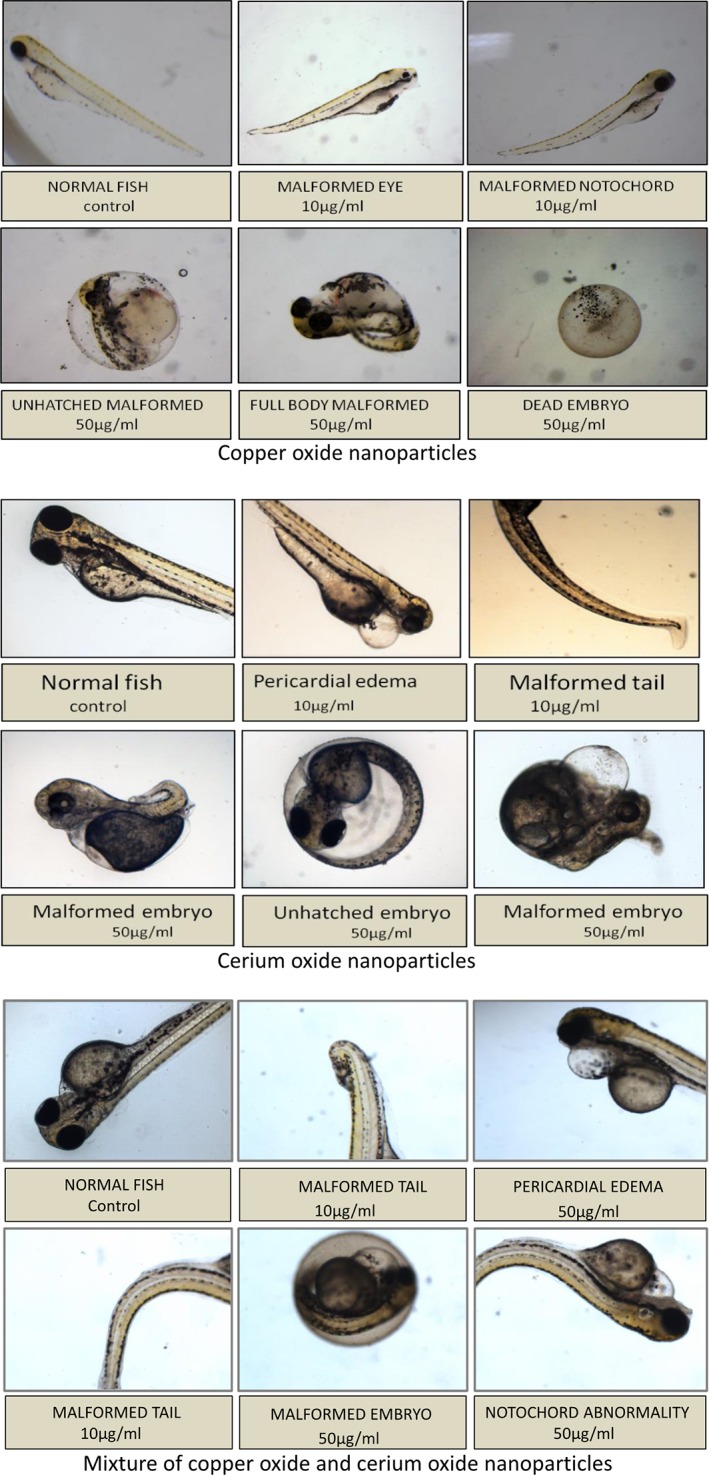
Malformations observed in zebra fish embryos treated with copper oxide nanoparticles, cerium oxide nanoparticles, and mixture of copper oxide and cerium oxide nanoparticles at 96 hpf [Color figure can be viewed at wileyonlinelibrary.com]

Malformations were observed in embryos treated with all the three groups of NP, and the number of malformed embryos also increased in a dose‐dependent and time‐dependent manner (Figure 2).

### 3.3 Effect of NP on cell viability

**Figure 3:**
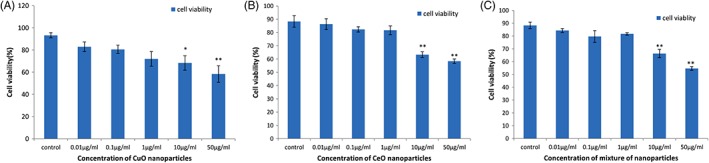
Viability of THP1 cells treated with (A) copper oxide nanoparticles, (B) cerium oxide nanoparticles, and (C) mixture of copper oxide and cerium oxide nanoparticles. Data are expressed as means ± SE from three independent experiments. Analysis of variance (α < 0.05), (*P < 0.05, **P < 0.01) [Color figure can be viewed at wileyonlinelibrary.com]

Cells survival rate in in vitro studies was assessed using trypan blue dye (Figure 3), and it was found that the survival rate of the cells treated with all the three cell lines decreased with increasing concentration and the lowest survival rate was found to be around 60% at the concentration of 50 μg/mL in case of CuO NP‐treated cells.

**Figure 4:**
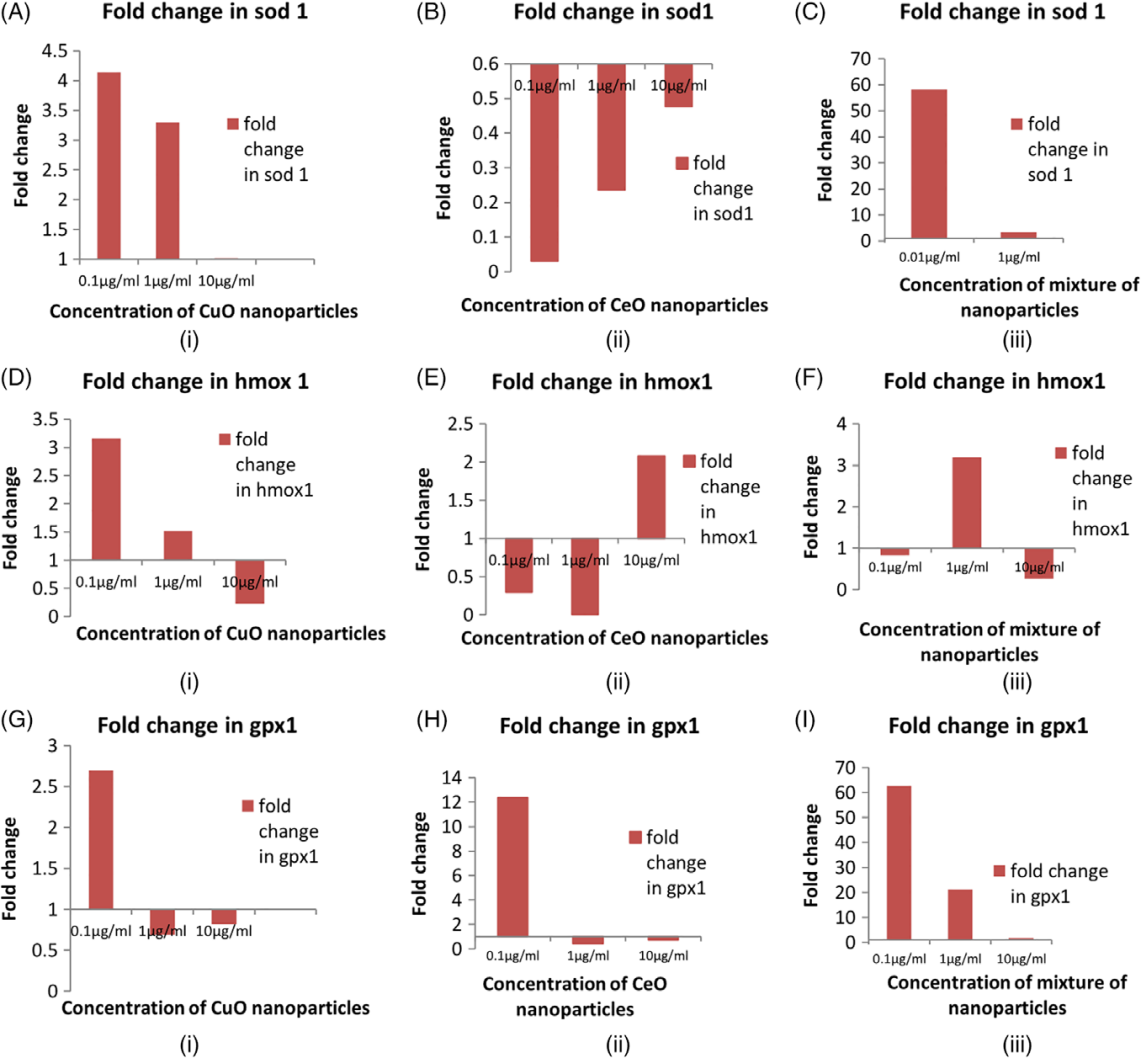
(A) Effect of nanoparticles on oxidative stress gene superoxide dismutase 1 (SOD1) in zebra fish treated with (i) copper oxide nanoparticles (ii) cerium oxide nanoparticles, and (iii) mixture of copper oxide and cerium oxide nanoparticles at 72 hpf. (B) Effect of nanoparticles on oxidative stress gene haem oxigenase 1 (HMOX1) in zebra fish treated with (i) copper oxide nanoparticles (ii) cerium oxide nanoparticles, and (iii) mixture of copper oxide and cerium oxide nanoparticles at 72 hpf. (C) Effect of nanoparticles on oxidative stress gene GPX1 in zebra fish treated with (i) copper oxide nanoparticles (ii) cerium oxide nanoparticles, and (iii) mixture of copper oxide and cerium oxide nanoparticles at 72 hpf [Color figure can be viewed at wileyonlinelibrary.com]

#### 3.3.1 Effect of NP on oxidative stress genes

The expression of three oxidative stress genes sod1, hmox1, and gpx1 was examined using total RNA isolated from 72 hpf embryos treated with the concentrations of 0.1 μg/mL, 1 μg/mL, and 10 μg/mL of different NP (Figure 4).

**Figure 5:**
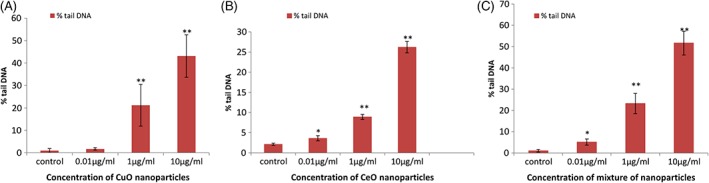
Comet assay of single‐cell suspension prepared from zebra fish embryos treated with (A) copper oxide nanoparticles, (B) cerium oxide nanoparticles, and (C) mixture of copper oxide and cerium oxide nanoparticles. Data are expressed as means ± SE from three independent experiments. Analysis of variance (*α* < 0.05), (*P < 0.05, **P < 0.01) [Color figure can be viewed at wileyonlinelibrary.com]

In case of CuO NP‐treated embryos, the expression of sod1 decreased with increasing concentrations with the downregulation of fourfolds at the concentration of 50 μg/mL, whereas the expression of hmox1 decreased up to 1 μg/mL. Similarly, the expression of gpx1 increased at lowest concentration, and then downregulation was observed at 1 μg/mL and 10 μg/mL.

In case of cerium oxide NP‐treated embryos, the expression of sod1 was downregulated at all concentrations, whereas the expression of hmox1 was first downregulated and then upregulation up to twofolds was observed at 50 μg/mL concentration. The expression of gpx1 was upregulated to about 12‐folds at 50 μg/mL concentration, whereas at other concentrations, downregulation of genes was observed.

In case of the embryos treated with the mixture of both the NP, the upregulation of sod1 decreased up to 1 μg/mL concentration and then there was a considerable increase of up to 176‐folds, whereas in case of hmox1, upregulation decreased in a concentration‐dependent manner. The expression of gpx1 first increased to about 62.68‐folds, and then, downregulation was observed to 1.6 at 50 μg/mL concentration.

#### 3.3.2 Effect of NP on catalase enzyme activity

The catalase activity was observed using NP‐treated zebra fish embryos at 96 hpf. In case of CuO NP, the catalase activity increased in a dose‐dependent manner to about 12 nmoL/mL/min up to 1 μg/mL concentration and then decrease in activity to about 7 nmoL/mL/min was observed in subsequent concentrations (Supporting Information Figure S3).

In case of cerium oxide NP and the mixture of these NP, not much variation was seen in the catalase activity of the treated embryos.

#### 3.3.3 DNA damage caused by NP

Single‐cell suspensions of zebra fish embryos treated with different concentrations of these NP were prepared, and comet assay was performed on these cells (Figure 5). It was found that the length of the tail of the comet increased in a dose‐dependent manner in case of CuO NP, cerium oxide NP, and the mixture of these NP. But more damage was found in case of CuO NP which accounted up to 100% at the concentration of 50 μg/mL. Whereas in case of cerium oxide NP and mixture of NP, the tail percentage at 50 μg/mL concentration was found to be 26.2% and 51.6%, respectively.

**Figure 6:**
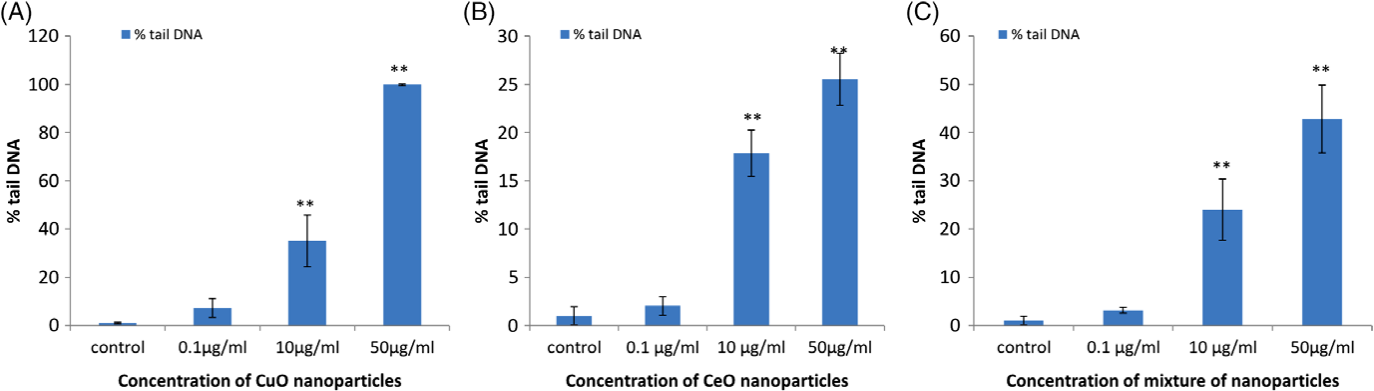
Comet assay of THP1 cells treated with (A) copper oxide nanoparticles, (B) cerium oxide nanoparticles, and (C) mixture of copper oxide and cerium oxide nanoparticles. Data are expressed as means ± SE from three independent experiments. Analysis of variance (α < 0.05), (*P < 0.05, **P < 0.01) [Color figure can be viewed at wileyonlinelibrary.com]

In case of in vitro studies (Figure 6), it was found that the percentage tail DNA was highest in case of CuO NP. It was found to be 100% at the concentration of 50 μg/mL. Whereas in case of cerium oxide NP and the mixture of these NP, the percentage tail DNA was found to be 25.52 and 43%, respectively, which signifies that highest damage to DNA was done at the highest concentrations of CuO NP.

#### 3.3.4 Effect of NP on apoptosis and necrosis

**Figure 7:**
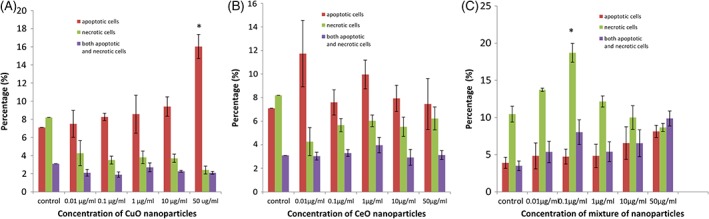
Percentage apoptosis and necrosis observed in single‐cell suspension prepared from zebra fish embryos at 96 hpf treated with (A) copper oxide nanoparticles, (B) cerium oxide nanoparticles, and (C) mixture of copper oxide and cerium oxide nanoparticles (not significant). Data are expressed as means ± SE from three independent experiments. Analysis of variance (α < 0.05), (*P < 0.05, **P < 0.01) [Color figure can be viewed at wileyonlinelibrary.com]

Single‐cell suspensions of zebra fish embryos treated with different concentrations of these NP were prepared, and apoptosis assay was performed on these cells (Figure 7). In case of CuO NP‐treated embryos, dose‐dependent increase in apoptosis was observed with the highest percentage of 16% at 50 μg/mL concentration, whereas no significant increase in necrosis was observed.

In case of cerium oxide NP, no significant increase in apoptosis and necrosis was observed, whereas in case of the mixture of NP, a very less increase was observed in apoptosis but the necrosis increased up to 18% at 0.1 μg/mL concentration, but then, a decrease was seen with increasing concentrations.

**Figure 8:**
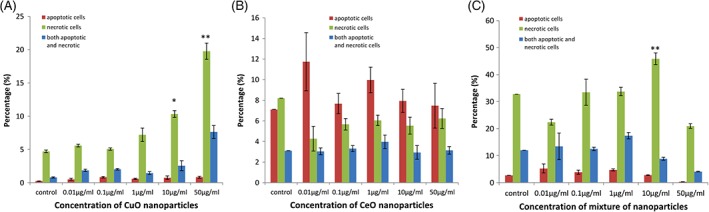
Percentage apoptosis and necrosis observed in THP1 cells treated with (A) copper oxide nanoparticles, (B) cerium oxide nanoparticles, and (C) mixture of copper oxide and cerium oxide nanoparticles (not significant). Data are expressed as means ± SE from three independent experiments. Analysis of variance (α < 0.05), (*P < 0.05, **P < 0.01) [Color figure can be viewed at wileyonlinelibrary.com]

In in vitro studies (Figure 8), in case of CuO NP, the percentage of necrotic cells increased at a considerable rate with increasing concentrations of NP up to 19.76% followed by the cells showing both necrosis and apoptosis. No significant increase in apoptosis was observed in the treated cells.

In case of cerium oxide NP, no significant increase in apoptosis and necrosis was observed in treated cells with increasing concentrations of NP, whereas in case of the cells treated with the mixture of these NP, considerable increase in necrotic cells were observed at 10 μg/mL concentration, but then, there was a drastic decrease at 50 μg/mL concentration. Similar was the case with the cells showing both apoptosis and necrosis, whereas no significant increase in apoptosis was observed in the cells with increasing concentrations of NP.

#### 3.3.5 Effect on cell survival

MTT assay was performed after treatment of the cells with different concentrations of NP, and percent survival of cells in comparison to the control cells without any treatment was calculated (Supporting Information Figure S4). It was found that percent survival of the cells decreased in a dose‐dependent manner. In case of CuO NP, the percent survival at 50 μg/mL concentration was found to be as low as 22%, whereas in case of cerium oxide NP and the mixture of these NP, the survival rate was found to be 88% and 68%, respectively.

#### 3.3.6 Cytokine release in THP‐1 cell line after NP treatment

**Figure 9:**
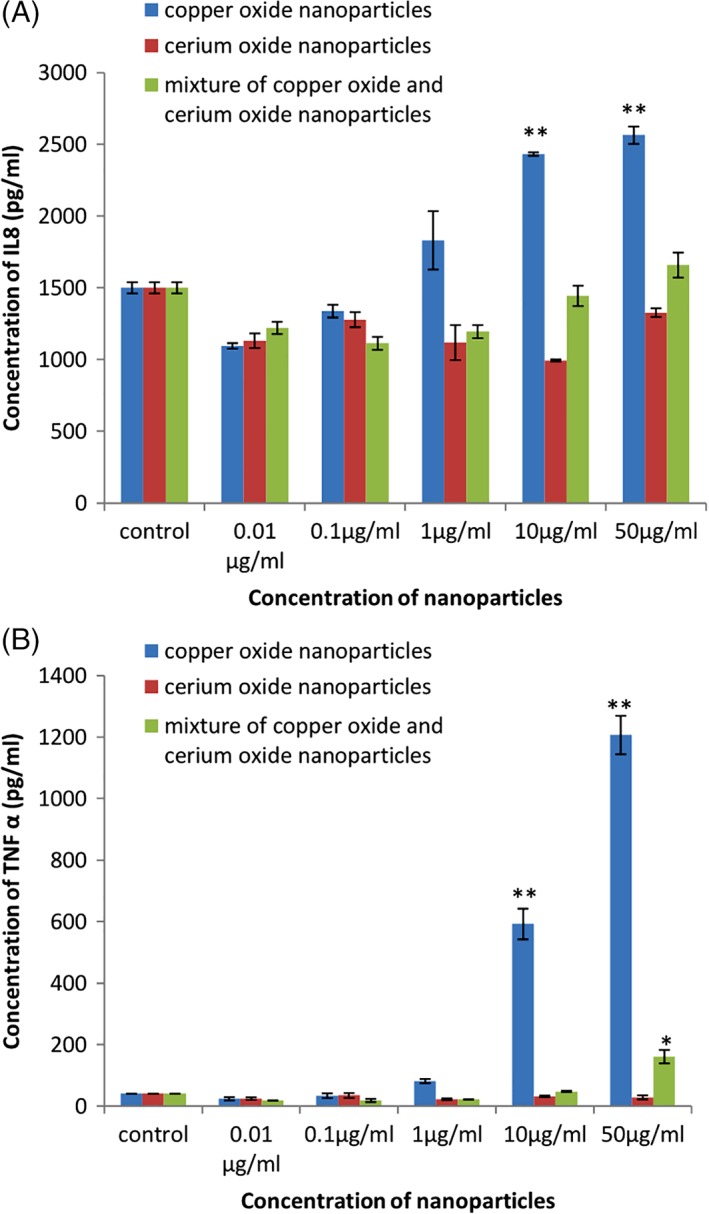
Percentage of cytokines (A) IL8 and (B) TNF‐α in THP 1 cells treated with copper oxide, cerium oxide nanoparticles, and mixture of nanoparticles. Data are expressed as means ± SE from three independent experiments. Analysis of variance (α < 0.05) [Color figure can be viewed at wileyonlinelibrary.com]

All the three types of NP had no effect on the concentration of IL 6 cytokines released by THP 1 cell lines. The concentration of two cytokines, namely IL 8 and TNF‐α, increased in a dose‐dependent and time‐dependent manner in case of CuO NP. The concentration of IL8 was 2464.38 pg/mL at 50 μg/mL concentration as compared to 1500.92 in control sample, and the concentration of TNF‐α was as high as 1206 pg/mL at 50 μg/mL concentration as compared to 40.355 pg/mL in control samples. No significant increase in concentration of both cytokines was observed in case of cerium oxide NP. In case of mixture of NP, significant increase in IL 8 and TNF‐α was observed only at the highest concentrations (Figure 9).

## 4 DISCUSSION

This study is an attempt to access the cytotoxic potential of NP, namely CuO NP and cerium oxide NP, and also to study the combined effect of these NP on zebra fish embryos as well as THP1 cell lines. Simple in vitro methods are relevant for initial toxicity testing, but physiologically relevant in vivo testing is necessary to better understand the toxicological mechanisms of different NP; therefore, in this study, both in vivo and in vitro models were used so that their results can be compared and evaluated efficiently. First, the characterization of NP was done in dry state as well as in the form of dispersions in Holtfreter's medium used for the development of fishes as well as RPMI 1640 medium used for the growth of THP 1 cell lines. Characterization of NP is necessary for toxicological studies as it influences the differential response of NP in biological systems.^23^ The XRD analysis showed that both the NP exist in crystalline form. The BET analysis for surface area showed that the surface area of CuO NP is 10 times higher than the cerium oxide NP, which may account for its higher toxicity. The DLS measurement and SEM images demonstrated that both CuO and cerium oxide NP had an average diameter of around 300‐400 nm when dispersed in both zebra fish and RPMI 1640. The zeta potential of the NP in dispersion form was found around −20 μE which confirmed the stability of NP in Holtfreter's medium. The ICP MS data showed that the concentration of copper ions and cerium ions was very less as compared to the CuO and cerium oxide NP thereby proving that the toxicity was caused by NP and not ions. In addition, results of ICP MS of the copper and cerium salts showed high concentrations of ions, but in those cases, the mortality and malformations also were not significant. This proved that the mortality of zebra fish is caused by the NP and not the ions.

The toxicity of these NP was then assessed on zebra fish embryos exposed to these NP at working concentrations of 0.01, 0.1, 1, 10, and 50 μg/mL. It is well documented that the developmental embryos are more sensitive to the external substances than the larval or adult stage. Therefore, the embryonic period was chosen to study the toxicity of zebra fish embryos. It was observed that with increase in concentration of CuO NP and the mixture of NP, the mortality of zebra fish embryos treated with the respective NP increased up to the concentration of 10 mg/L and then decreased. This decrease in mortality of zebra fish at 50 μg/mL concentration might be observed due to the aggregation of NP at higher concentrations. According to the literature, it has been speculated that cerium oxide nanomaterials form less aggregates as compared to other nanomaterials.^24^ It was observed that the size of cerium oxide after dispersion into medium was found to be around 172 nm, which is less as compared to CuO nanomaterials at 50 μg/mL.^25^ Earlier studies have also indicated that NP generally aggregate at higher concentrations.^26^ It was also reported that silver NP formed aggregates that were not able to enter the chorion and got deposited on the surface. Therefore, less mortality rate was observed at 50 μg/mL concentration.^26^ No such pattern was observed in case of cerium oxide NP, which might be due to their less aggregation as compared to CuO NP. Moreover, less mortality was observed in case of cerium oxide NP indicating the lower toxicity of cerium oxide NP as compared to CuO NP and the mixture of these NP which may be attributed to the high surface area of CuO NP. We attempted to perform the same toxicological assay on zebra fish embryos after removing their chorion with the help of pronase enzyme. It was found that all the embryos died after 72 hpf after treatment with these NP thereby proving that chorion protects the embryos from copper NP to some extent. Because no change in mortality rate and body morphology was found in case of zebra fishes treated with copper ions and cerium ions only, it was evident that the mortality and malformations are caused by CuO nanomaterials and not by copper ions.

The hatching rate of zebra fish embryos was found to decrease in case of CuO NP and mixture of NP. During normal process of hatching of embryos, chorion is digested by the hatching enzymes secreted from hatching gland cells of embryos (Sreedevi, 2014). Therefore, hatching was delayed in case of embryos treated with CuO NP and the mixture of these NP due to disturbance of this enzyme by copper ions released from CuO NP. Cerium oxide NP had a very less effect on the hatching rate of zebra fish embryos.

Various malformations such as pericardial edema, notochord abnormality, eye malformation, and tail malformations were observed in treated embryos with the highest number of malformations observed in case of CuO NP treated zebra fish embryos. Although in case of cerium oxide NP and the mixture of NP, the malformations were comparatively less. These malformations occur due to reactive oxygen species (ROS) production which further leads to the oxidative damage in fishes.^27^

Trypan blue staining of the THP‐1 cells showed that cell viability decreased up to 60% in all three kinds of NP, and these results are very similar to the mortality rate of zebra fish embryos treated with these NP. This implies that these NP are toxic to cell lines too at comparable doses which were given to zebra fish embryos. Similar results were observed when K562 cells were treated with CuO NP at a concentration of 25 μg/mL.^10^ The toxicity of cerium NP was also confirmed on the treatment of human neuroblastoma cells, which resulted in cell death of around 60% cells.^43^

RT‐PCR analysis of different genes involved in oxidative stress, namely sod 1, hmox 1, and gpx 1, was done in the treated zebra fish embryos. These genes were seen to be downregulated in case of CuO NP, whereas only sod 1 was found to be downregulated in case of cerium oxide NP. In case of the mixture of these NP, all genes were upregulated at first, but then, again downregulation was observed at 50 μg/mL concentrations. Downregulation of these genes was observed in case of zinc oxide NP‐treated zebra fish embryos.^28^ The downregulation of genes might be occurring due to the deleterious effects of metals on DNA as it was reported in previous studies that the expression of antioxidant genes were modulated in metals exposed fishes, bivalves and protozoa's.^29^ This shows that nanomaterials are disturbing the normal mechanisms of oxidative stress the zebra fish embryos in a deleterious fashion, which further exhibit toxic effect of these nanomaterials.

Catalase enzyme is involved in detoxification of hydrogen peroxide, a ROS, and its increased enzyme activity is directly proportional to increased production of ROS in the body which further leads to toxic effects in the body.^30^ It was found that with increasing concentration of CuO NP, the catalase activity increased and then a decline was observed. The increased activity implies the increased production of ROS thereby inferring increased toxicological effects. In case of zebra fish embryos treated with cerium oxide NP and the mixture of these NP, not much variation was seen in the catalase activity. The uneven pattern that was observed in case of CuO NP might be due to the interaction of NP and proteins. NP and proteins are found to interact with various macromolecules, and these interactions may also affect the outcome of spectrophotometric assays. NP are found to interfere with spectrophotometric assays due to high adsorption capacity and optical activity. Even these NP are found to bind to dyes such as MTT dye and alter their structure. The range and type of interference depend on many factors such as coating, biological media, and concentration. Repeated washes are also not able to remove NP as these nanomaterials are found to enter cells and attach to membrane.^14^, ^31^

DNA damage which can either occur by direct interaction of particles with genetic material or by secondary damage from particle‐induced ROS which can be further confirmed by comet assay.^44^ It is well documented that transition metal NP induce chromosomal aberrations, mutations, and DNA strand breaks.^32^ The free radicles formed by oxidative stress caused by nanomaterials react with all the components of DNA causing single‐stranded DNA breaks in DNA.^33^ Similar results were found when zebra fish embryos were treated with different concentrations of CuO NP. The tail of DNA comet represents the extent of damage done to the DNA, and it was found that the tail of the comet was largest in case of CuO NP, whereas less DNA damage was observed in cerium oxide NP and the mixture of NP. Similar results were found in in vitro studies too.

Apoptosis is considered as the major mechanism of cell death caused by the NP‐induced oxidative stress.^34^ CuO NP are found to induce ROS‐mediated cell death via mitochondrial dysfunction.^35^ Therefore, single‐cell suspension prepared from zebra fish embryos treated with CuO NP show maximum apoptosis, whereas no significant apoptosis and necrosis was observed in case of single‐cell suspension prepared from zebra fish embryos treated with cerium oxide NP. In case of single‐cell suspension prepared from zebra fish embryos treated with mixture of these NP, less apoptosis was observed, whereas necrosis first increased and then decreased considerably. This might be due to the interaction of NP with PI (propidium iodide) which has already been reported earlier, which overrepresents the results in detection of necrotic cells.^36^ Similarly, in in vitro studies, it was found that in cells treated with CuO NP, the necrosis of cells increased in a dose‐dependent manner whereas no significant effect was observed in the apoptosis of cells. These results are totally opposite to the ones obtained in case of single‐cell suspension of zebra fish embryos treated with CuO NP. Earlier studies suggest that CuO NP cause cell death by apoptosis.^37^ Cells treated with cerium oxide NP did not show significant apoptosis or necrosis as discussed in previous studies.^24^ The cells treated with the mixture of NP also did not show any significant increase in apoptosis.

MTT assay was also performed, and it was observed that, in case of cells treated with CuO NP, the survival rate was found to be 22% only in 50 μg/mL concentration with a dose‐dependent decrease. Reference 38 also observed similar results in his studies of the effect of CuO NP on THP1 cells. In case of cells treated with cerium oxide NP, the cell viability also increased in a dose‐dependent manner and it was as high as 88% at 50 μg/mL concentration thus indicating its low toxicity again. In case of cells treated with the mixture of NP, survival rate was found to be 68% in the highest concentration with a dose‐dependent decrease. This also displays that CuO NP are the most toxic ones in this group.

NP interact with various components of the immune system and leads to either enhancement or inhibition of its functions.^39^, ^40^ It has been studied that certain nanomaterials can be immunotoxic, although no description of standard immunotoxicity assays have been given so far.^40^ Evaluation of immunotoxicity of NP can be done by measuring the level of pro‐inflammatory cytokines. High level of cytokines after treatment with nanomaterials signifies their toxicity. In our study, we evaluated the effect of NP on the release of pro‐inflammatory cytokines namely IL6, IL8, and TNF α. The concentration of IL6 was not affected by any of the NP. The maximum concentration of IL6 was found to be around 65 pg/mL in cells treated with 10 μg/mL of CuO nanomaterials which was comparable to the control with 55 pg/mL concentration. Similar results were seen with cerium oxide and mixture of these nanomaterials. Comparable results were observed when primary macrophages were treated with CuO nanomaterials.^41^ IL8 concentration in THP 1 cells treated with CuO NP was found to increase significantly in a dose‐dependent manner, that is, up to 2500 pg/mL at a concentration of 50 μg/mL. IL8 leads to the recruitment of neutrophils to the site of damage and, therefore, implying that CuO nanomaterials lead to cellular damage in cells. In previous studies, the level of IL8 was found to increase twofolds when Caco 2 cells were treated with CuO NP.^42^, ^44^ No significant effect on IL8 was observed in case of cerium oxide NP and the mixture of these NP. The concentration of TNF α increased significantly in a dose‐dependent manner in case of THP 1 cells treated with CuO NP, that is, around 1200 pg/mL at the highest concentration. Similar effect was observed when zebra fish cells were treated with CuO NP.^18^ No significant increase was found in THP 1 cells treated with cerium oxide NP, whereas the level of TNFα was also found to increase significantly, that is, up to 200 pg/mL at the highest concentration of mixture of nanomaterials. This signifies that CuO NP lead to increase in the concentration of pro‐inflammatory cytokines therefore implying the immunotoxic effect of these NP.

## 5 CONCLUSION

In the present study, concentrations of NP ranging from very low to high were used to access their toxic effects on zebra fish embryos and cell lines. It was found in our studies that CuO NP cause severe harmful effects on the development of zebra fish embryos as compared to cerium oxide NP. In case of mixture of these NP, the harmful effects were more than cerium oxide NP and less than that of CuO NP. Therefore, synergistic effect was not observed in case of mixture of these NP. Studies using THP1 cell lines were also performed, and the results obtained were comparable to that obtained by studies with zebra fish embryos but comprehensive studies were only possible with zebra fish embryos.

Further studies are required to understand the pattern of trafficking of these NP, and also different mechanisms involved in causing toxicological effects in zebra fish embryos. The chronic effects of these NP also need to be investigated to observe the long‐term effects of these NP on living beings.

## CONFLICT OF INTEREST

There are no conflicts of interest regarding the publication of this article.

## Supporting information

Figure S1 SEM images of (a) copper oxide nanoparticles and (b) cerium oxide nanoparticles.

Figure S2 Percentage hatching in zebra fish embryos treated with (a) copper oxide nanoparticles, (b) cerium oxide nanoparticles, and (c) mixture of copper oxide and cerium oxide nanoparticles. Data are expressed as means ± SE from three independent experiments. Analysis of variance (α < 0.05)

Figure S3 Catalase activity observed in zebra fish embryos treated with copper oxide nanoparticles at 96 hpf. Data are expressed as means ± SE from three independent experiments. Analysis of variance (α < 0.05)

Figure S4 Percent survival in THP1 cells treated with (a) copper oxide nanoparticles, (b) cerium oxide nanoparticles (not significant), and (c) mixture of copper oxide and cerium oxide nanoparticles. Data are expressed as means ± SE from three independent experiments. Analysis of variance (α < 0.05)

Figure S5 Images of comets produced after doing comet assay.

Figure S6 XRD images of (a) copper oxide nanoparticles and (b) cerium oxide nanoparticles in dry form.
