# Supplementary figures and images for: Toxicological evaluation of metal oxide nanoparticles and mixed exposures at low doses using zebra fish and THP1 cell line
Source: Environ Toxicol. 2018 Dec 12;34(4):375–87. doi: 10.1002/tox.22692 (PMC6492081; doi:10.1002/tox.22692)

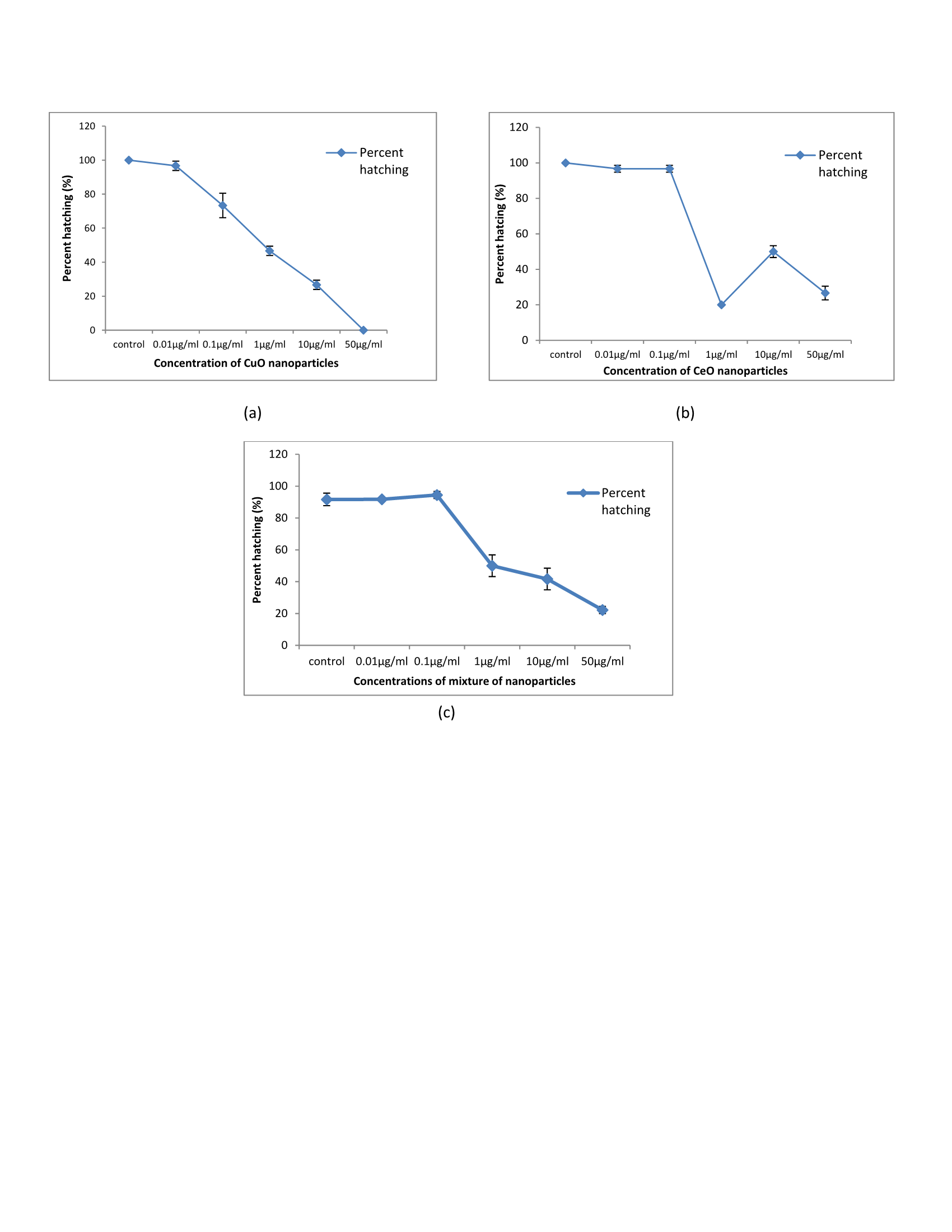

Supplement: Supplementary file 2 — Figure S2 Percentage hatching in zebra fish embryos treated with (a) copper oxide nanoparticles, (b) cerium oxide nanoparticles, and (c) mixture of copper oxide and cerium oxide nanoparticles. Data are expressed as means ± SE from three independent experiments. Analysis of variance (α < 0.05) [file TOX-34-375-s002.TIF]

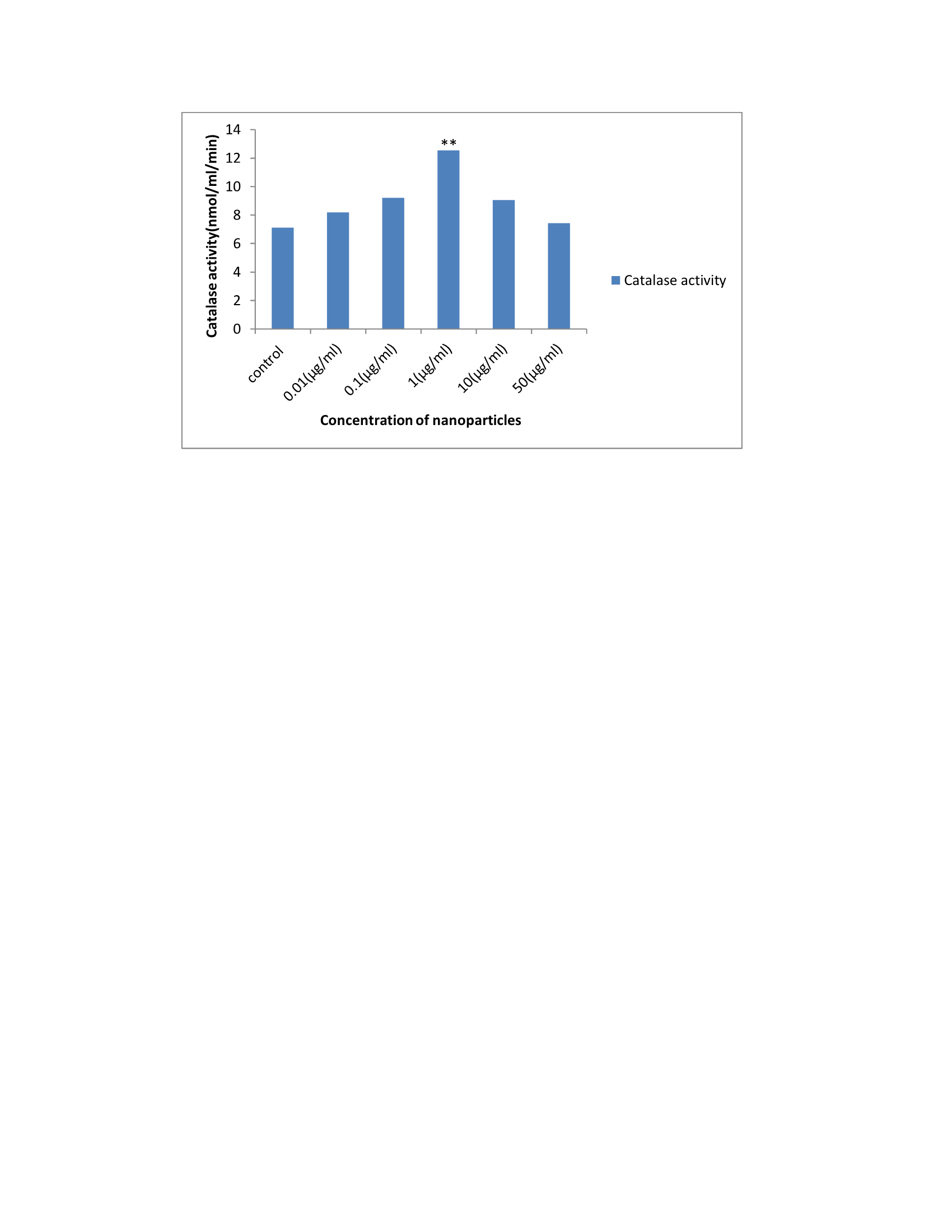

Supplement: Supplementary file 3 — Figure S3 Catalase activity observed in zebra fish embryos treated with copper oxide nanoparticles at 96 hpf. Data are expressed as means ± SE from three independent experiments. Analysis of variance (α < 0.05) [file TOX-34-375-s003.TIF]

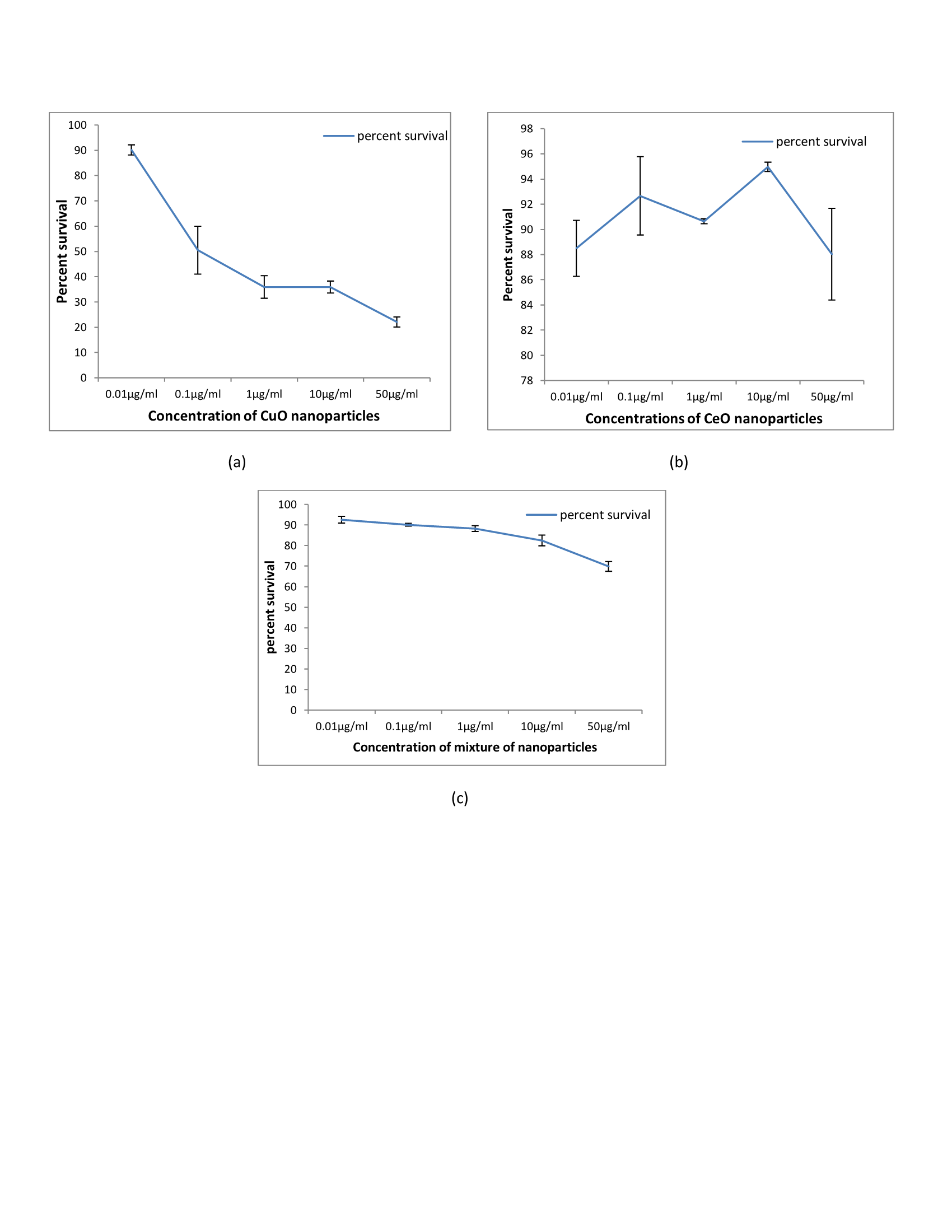

Supplement: Supplementary file 4 — Figure S4 Percent survival in THP1 cells treated with (a) copper oxide nanoparticles, (b) cerium oxide nanoparticles (not significant), and (c) mixture of copper oxide and cerium oxide nanoparticles. Data are expressed as means ± SE from three independent experiments. Analysis of variance (α < 0.05) [file TOX-34-375-s004.TIF]

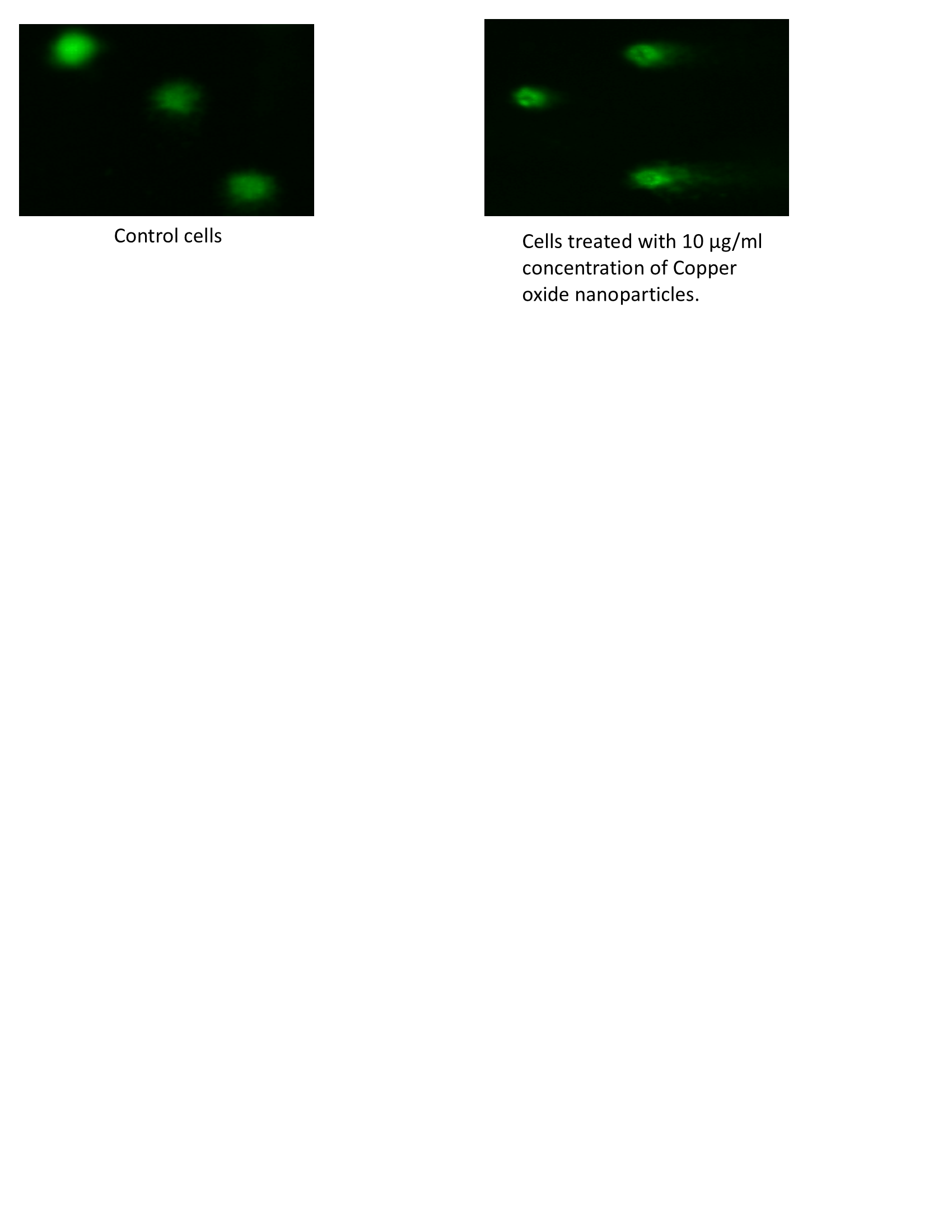

Supplement: Supplementary file 5 — Figure S5 Images of comets produced after doing comet assay. [file TOX-34-375-s005.TIF]

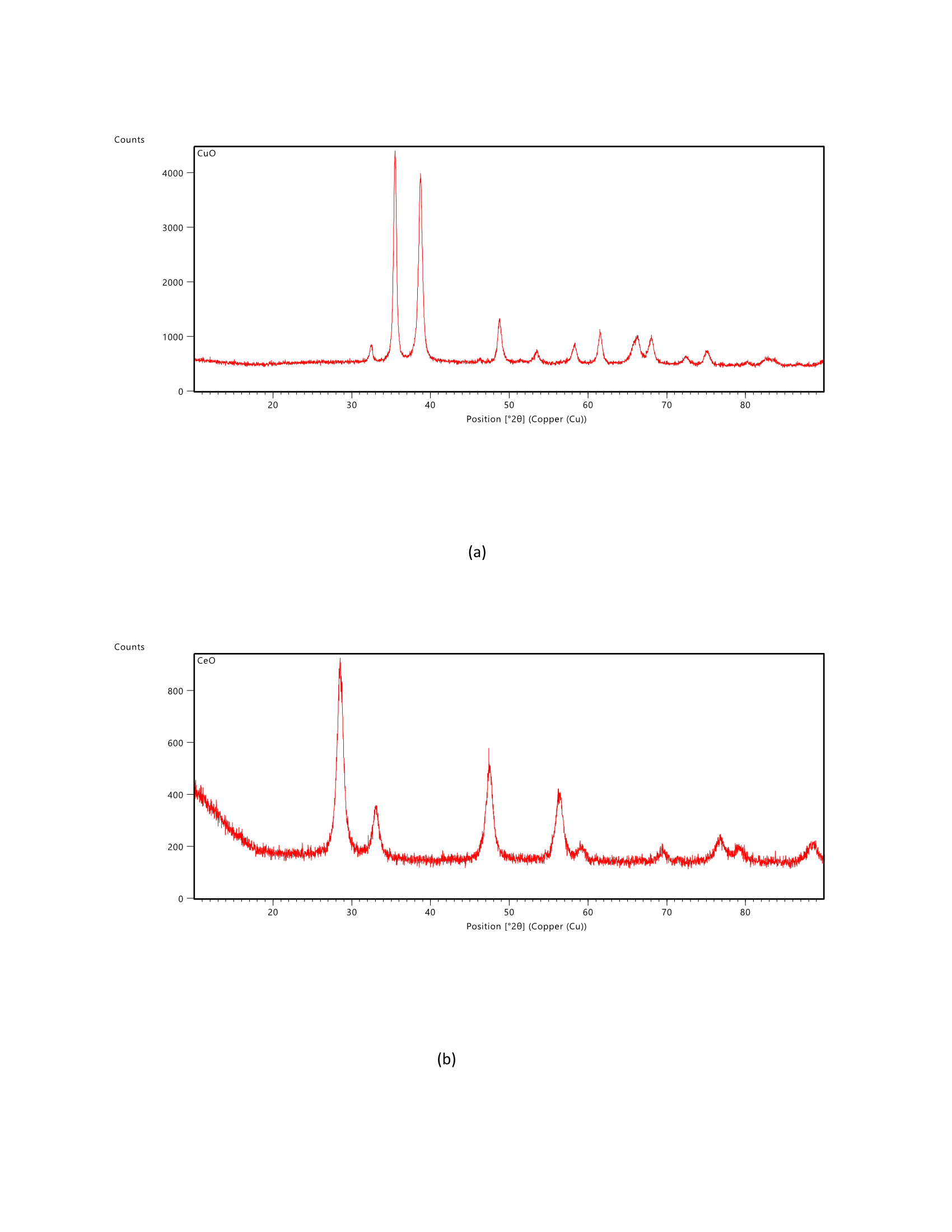

Supplement: Supplementary file 6 — Figure S6 XRD images of (a) copper oxide nanoparticles and (b) cerium oxide nanoparticles in dry form. [file TOX-34-375-s006.TIF]
